# CGRP Regulates the Age-Related Switch Between Osteoblast and Adipocyte Differentiation

**DOI:** 10.3389/fcell.2021.675503

**Published:** 2021-05-26

**Authors:** Hang Li, Jian Qu, Haihong Zhu, Jiaojiao Wang, Hao He, Xinyan Xie, Ren Wu, Qiong Lu

**Affiliations:** ^1^Department of Pharmacy, The Second Xiangya Hospital of Central South University, Changsha, China; ^2^Institute of Clinical Pharmacy, Central South University, Changsha, China; ^3^Department of Vascular Surgery, The Second Xiangya Hospital of Central South University, Changsha, China; ^4^Department of Orthopedics, The Second Xiangya Hospital of Central South University, Changsha, China

**Keywords:** CGPR, osteoporosis, BMSCs, osteogenic, adipogenic, age-related

## Abstract

Osteoporosis is a chronic age-related disease. During aging, bone marrow-derived mesenchymal stem cells (BMSCs) display increased adipogenic, along with decreased osteogenic, differentiation capacity. The aim of the present study was to investigate the effect of calcitonin gene-related peptide (CGRP) on the osteogenic and adipogenic differentiation potential of BMSC-derived osteoblasts. Here, we found that the level of CGRP was markedly lower in bone marrow supernatant from aged mice compared with that in young mice. *In vitro* experiments indicated that CGRP promoted the osteogenic differentiation of BMSCs while inhibiting their adipogenic differentiation. Compared with vehicle-treated controls, aged mice treated with CGRP showed a substantial promotion of bone formation and a reduction in fat accumulation in the bone marrow. Similarly, we found that CGRP could significantly enhance bone formation in ovariectomized (OVX) mice *in vivo*. Together, our results suggested that CGRP may be a key regulator of the age-related switch between osteogenesis and adipogenesis in BMSCs and may represent a potential therapeutic strategy for the treatment of age-related bone loss.

## Introduction

Osteoporosis is a chronic disease caused by the breakdown of bone homeostasis, with elderly and postmenopausal women being the populations most at risk of developing this condition (NIH Consensus Development Panel on Osteoporosis Prevention, Diagnosis, and Therapy, 2001; Rachner et al., 2011). Age-related bone loss and osteoporosis have been associated with reduced numbers of osteoblasts and increased numbers of adipocytes (Idris et al., 2009; Yu and Wang, 2016). It is known that bone marrow mesenchymal stem cells (BMSCs) have the potential to differentiate into osteoblasts, adipocytes, and osteoclasts, thereby playing an important role in bone formation (Li et al., 2016; Chen et al., 2018; Peng et al., 2019). Overall, bone homeostasis depends on the balance between the osteogenic and adipogenic differentiation of BMSCs (Li et al., 2015; Lv et al., 2018). Age-related osteoporosis results from reduced osteogenic differentiation and increased adipogenic differentiation of BMSCs in elderly patients (Shen et al., 2012; Childs et al., 2015; Li et al., 2017).

Calcitonin gene-related peptide (CGRP), a member of the calcitonin protein family, is a 37-amino acid peptide generated through the alternative splicing of primary transcripts of the calcitonin gene (Amara et al., 1982; Rosenfeld et al., 1983; Naot and Cornish, 2008). In humans and mice, CGRP exists as two isoforms—α-CGRP and β-CGRP—which are encoded by the *CALCA* and *CALCB* genes, respectively. In mice, α-CGRP is highly expressed in the central nervous system and peripheral nervous system, while β-CGRP is expressed in the enteric nervous system (Sternini, 1992; Russell et al., 2014). In addition, in mice, the *Calca* gene has been shown to affect bone remodeling; however, the underlying mechanism remains unclear (Schinke et al., 2004).

CGRP is a neuropeptide that is released from sensory nerve endings and can also be found in bone cells and endothelial cells (Russell et al., 2014). There is evidence that CGRP is involved in the regulation of cell proliferation and differentiation, and may also be important for connecting the systems involved in bone metabolism (Bjurholm et al., 1988; Chattergoon et al., 2005; Thievent et al., 2005; Zhang et al., 2016; Xu et al., 2019). Additionally, studies have confirmed that CGRP can promote osteogenesis and inhibit osteoclast formation (Imai et al., 1997; Villa et al., 2003; Yoo et al., 2014). However, few studies have investigated the roles of CGRP in the age-related switch between osteoblast and adipocyte differentiation in bone marrow. Consequently, the aim of this study was to investigate whether CGRP is associated with increased bone formation and inhibition of adipocyte accumulation in age-related bone loss.

In the current study, we show that the level of CGRP in mouse bone marrow-derived supernatant decreases with aging. Furthermore, we found that CGRP not only promotes the osteogenic differentiation of BMSCs but also inhibits their adipogenic differentiation and senescence. Importantly, we demonstrate that exogenous application of CGRP can accelerate bone formation in aged and OVX mice *in vivo*, implying that CGRP may be a potential therapeutic target for the prevention of osteoporosis.

## Materials and Methods

### Mice

C57BL/6JN mice were purchased from Hunan Slaccas Jingda (Changsha, China). Two-month-old female C57BL/6JN mice underwent bilateral ovarian resection to establish the OVX model. For *in vitro* CGRP treatment experiments, 12-month-old OVX mice and sham-operated mice were injected with CGRP (10 mg/kg) *via* the tail vein three times weekly for 1 month. Mice treated with 1 × PBS were used as controls. All mice were maintained in the specific-pathogen-free facility of the Laboratory Animal Research Center of Central South University. The mice were kept under a 12 h/12 h light: dark cycle and had adequate access to food and water. All animal care protocols and experiments were reviewed and approved by the Animal Care and Use Committee of the Laboratory Animal Research Center at The Second Xiangya Hospital of Central South University.

### Isolation and Culture of BMSCs

BMSCs were isolated as previously described (Picke et al., 2018). Briefly, the femora and tibiae of four 7-day-old mice were cut and digested with Liberase DL (26 U/mL) (Roche) in a water bath at 37°C for 2 h. The cell suspension was centrifugated at 1,000 rpm for 5 min at 4°C, resuspended in 1 mL of α-MEM containing 1% penicillin/streptomycin and 10% FBS, and then cultured in a 10-cm culture dish. The medium was replaced every other day and CD11b^+^ cells were cleared using anti-CD11b antibody-coated magnetic beads.

### Osteogenic Differentiation and Mineralization Assay

Isolated BMSCs were digested with 0.25% trypsin and diluted to 1 × 10^7^ cells/mL. Then, 200 μL of the cell suspension was plated in 6-well plates at a density of 2 × 10^6^ cells/well. At 80% confluence, the medium was replaced with osteogenic induction medium containing 10% FBS, 1% penicillin/streptomycin, 0.1 mM dexamethasone, 10 mM β-glycerol phosphate, and 50 mM ascorbate. The osteogenic induction medium was renewed every 3 days for 21 days. The cells were subsequently fixed in 4% paraformaldehyde and stained with 2% Alizarin Red S (Sigma-Aldrich). The Alizarin Red S was dissolved in cetylpyridinium chloride solution and quantified by spectrophotometry at 540 nm.

### Adipogenic Differentiation Assay

Isolated BMSCs were plated in 6-well plates at 2.5 × 10^6^ cells/well in adipogenic differentiation medium containing 10% FBS, 1% penicillin/streptomycin, 0.5 mM 3-isobutyl-1-methylxanthine, 1 μM dexamethasone, and 5 μg/mL insulin. The adipogenic induction medium was renewed every 3 days for 14 days. The cells were then fixed in 4% paraformaldehyde and stained with Oil Red solution (Sigma-Aldrich). The dye was solubilized in isopropanol and the absorption at 540 nm was measured using a BioTek Epoch microplate spectrophotometer (BioTek Instruments).

### Enzyme-Linked Immunosorbent Assay (ELISA)

Bone marrow was separated, centrifuged at 1,000 rpm for 10 min, and then the supernatant was transferred to a new centrifuge tube. ELISA was performed using a CGPR (rat, mouse) EIA-Kit (K-01509, Phoenix Pharmaceuticals) according to the manufacturer’s instructions.

### Quantitative Real-Time PCR Analysis

Total RNA (1 μg) isolated from CGRP-treated BMSCs was treated with gDNA Eraser to remove residual genomic DNA and then reverse-transcribed into cDNA using the PrimeScript RT reagent Kit (Takara, Japan). Real-time PCR analysis was performed with SYBR Green (Takara, Japan) in an Applied Biosystems QuantStudio 3 Real-Time PCR System (Applied Biosystems). The amplification reactions were performed in a 96-well plate and consisted of 40 cycles of 95°C for 5 s and 60°C for 30 s. The relative transcript levels of target genes were quantified using the 2^–△^^△^^*Ct*^ method with beta-actin serving as the internal control. The primer sequences are listed in Table 1.

**TABLE 1:** Primer pairs in this study.

**Name**	**Sequence (5′-3′)**	**Amplicon size (bp)**
Gdpd2-F	CCAGCAAGTGCGACTGTATCT	185
Gdpd2-R	GACCAGGAGAGAGACGACCA	
Igf1-F	CTGGACCAGAGACCCTTTGC	269
Igf1-R	GGACGGGGACTTCTGAGTCTT	
Fgf9-F	ATGGCTCCCTTAGGTGAAGTT	104
Fgf9-R	TCATTTAGCAACACCGGACTG	
Wnt10b-F	GCGGGTCTCCTGTTCTTGG	71
Wnt10b-R	CCGGGAAGTTTAAGGCCCAG	
Gli2-F	GGGACTCTTTAGCCTCGCAG	158
Gli2-R	CCACAGGGTTGAGGTAGTCAT	
Rspo2-F	CCAAGGCAACCGATGGAGAC	100
Rspo2-R	TCGGCTGCAACCATTGTCC	
Jag1-F	CCTCGGGTCAGTTTGAGCTG	150
Jag1-R	CCTTGAGGCACACTTTGAAGTA	
Sox2-F	GCGGAGTGGAAACTTTTGTCC	157
Sox2-R	CGGGAAGCGTGTACTTATCCTT	
Igfbp3-F	CCAGGAAACATCAGTGAGTCC	101
Igfbp3-R	GGATGGAACTTGGAATCGGTCA	
Igf2-F	GTGCTGCATCGCTGCTTAC	222
Igf2-R	ACGTCCCTCTCGGACTTGG	
Bmp8a-F	AGTCTCTGGTCAGTACCACAG	160
Bmp8a-R	TGTTTACGCAGGATGACATTGTT	
Bmp4-F	ATTCCTGGTAACCGAATGCTG	89
Bmp4-R	CCGGTCTCAGGTATCAAACTAGC	
Id4-F	CAGTGCGATATGAACGACTGC	72
Id4-R	GACTTTCTTGTTGGGCGGGAT	
Bmp6-F	AGAAGCGGGAGATGCAAAAGG	211
Bmp6-R	GACAGGGCGTTGTAGAGATCC	
Acvr2a-F	GCGTTCGCCGTCTTTCTTATC	108
Acvr2a-R	GTTGGTTCTGTCTCTTTCCCAAT	
Pth1r-F	CAGGCGCAATGTGACAAGC	125
Pth1r-R	TTTCCCGGTGCCTTCTCTTTC	
Lrp5-F	ACGTCCCGTAAGGTTCTCTTC	172
Lrp5-R	GCCAGTAAATGTCGGAGTCTAC	
Hdac5-F	AGCACCGAGGTAAAGCTGAG	91
Hdac5-R	GCTGTGGGAGGGAATGGTT	
Hey1-F	GCGCGGACGAGAATGGAAA	231
Hey1-R	TCAGGTGATCCACAGTCATCTG	
Dlk1-F	AGTGCGAAACCTGGGTGTC	148
Dlk1-R	GCCTCCTTGTTGAAAGTGGTCA	
Tmem119-F	CCTACTCTGTGTCACTCCCG	212
Tmem119-R	CACGTACTGCCGGAAGAAATC	
Fam20c-F	GATGTGACGCGGGATAAGAAG	100
Fam20c-R	GCTCGGTGGAACAGTAGTAGG	
Gja1-F	ACAGCGGTTGAGTCAGCTTG	106
Gja1-R	GAGAGATGGGGAAGGACTTGT	
Tgfbr3-F	GGTGTGAACTGTCACCGATCA	125
Tgfbr3-R	GTTTAGGATGTGAACCTCCCTTG	
Col1a1-F	GCTCCTCTTAGGGGCCACT	103
Col1a1-R	CCACGTCTCACCATTGGGG	
Ift80-F	AGCTGTGTGGGTTGGACTAC	107
Ift80-R	AGCTTGACTATTAGGCTGGTTTC	
Hmga2-F	GAGCCCTCTCCTAAGAGACCC	106
Hmga2-R	TTGGCCGTTTTTCTCCAATGG	
Ptgs2-F	TGTGACTGTACCCGGACTGG	233
Ptgs2-R	TGCACATTGTAAGTAGGTGGAC	
Vgf-F	AAGGATGACGGCGTACCAGA	114
Vgf-R	TGCCTGCAACAGTACCGAG	
Per2-F	GAAAGCTGTCACCACCATAGAA	101
Per2-R	AACTCGCACTTCCTTTTCAGG	
Acads-F	TGGCGACGGTTACACACTG	231
Acads-R	GTAGGCCAGGTAATCCAAGCC	
Fabp3-F	ACCTGGAAGCTAGTGGACAG	106
Fabp3-R	TGATGGTAGTAGGCTTGGTCAT	
Prkab2-F	ACCATCTCTATGCACTGTCCA	86
Prkab2-R	CAGCGTGGTGACATACTTCTT	
Dgat1-F	TCCGTCCAGGGTGGTAGTG	199
Dgat1-R	TGAACAAAGAATCTTGCAGACGA	
Hdac6-F	TCCACCGGCCAAGATTCTTC	109
Hdac6-R	CAGCACACTTCTTTCCACCAC	
Egr2-F	GCCAAGGCCGTAGACAAAATC	154
Egr2-R	CCACTCCGTTCATCTGGTCA	
Apmap-F	CCTTGCCATTCCCCTACTTGG	116
Apmap-R	ACTTCGTATTTGGATGCAGAACA	
FABP4-F	AAGGTGAAGAGCATCATAACCCT	133
FABP4-R	TCACGCCTTTCATAACACATTCC	
PPARGγ-F	TCGCTGATGCACTGCCTATG	103
PPARGγ-R	GAGAGGTCCACAGAGCTGATT	
β-actin-F	GGCTGTATTCCCCTCCATCG	154
β-actin-R	CCAGTTGGTAACAATGCCATGT	

### Micro-CT Analysis

Micro-computed tomography (micro-CT) analysis was performed as previously reported (Li et al., 2015; Yang et al., 2019). The femora of mice from both the CGRP treatment group and the control group were isolated, fixed in 4% formaldehyde for 24 h, and then scanned by X-ray microtomography (Skyscan 1172, Bruker) at a pixel size of 13.98 μm. For the distal femur, the region-of-interest (ROI) was defined from 0.215 to 1.72 mm below the growth plate. The bone volume as a fraction of total bone volume (BV/TV), trabecular thickness (Tb. Th), trabecular number (Tb. N), and trabecular separation (Tb. Sp) were measured.

### Immunohistochemistry and Tartrate-Resistant Acid Phosphatase (TRAP) Staining

The femora were decalcified with 0.5 M EDTA for 1–2 weeks and then embedded in paraffin. The paraffin-embedded femora were sectioned (4 μm) using a RM2135 rotary microtome (Leica Geosystems). Sections were roasted at 60°C in an oven for 2 h, dewaxed with dimethyl benzene, dehydrated with alcohol, and treated with an antigen retrieval solution. The sections were subsequently washed with 1 × TTBS, blocked with 5% goat serum, and incubated with an anti-osteocalcin primary antibody (diluted 1:500; Cat# M041, Takara) overnight at 4°C. The next day, the sections were washed with 1 × TTBS, incubated with biotinylated secondary antibody (anti-mouse,1:200; Cat# PV9000, Beijing Zhongshan Jinqiao Biotechnology Co. Ltd.) for 1 h, washed with 1 × TTBS, and counterstained with hematoxylin (Sigma–Aldrich) for immunohistochemical analysis. An inverted fluorescence microscope was used for imaging. For TRAP staining, an Osteoclast Staining Kit (Sigma–Aldrich) was used according to the manufacturer’s instructions. The number of osteoblasts on the bone surface and osteoclast number per bone perimeter were measured in the femora.

### β-Galactosidase Staining

BMSCs were washed with 1 × PBS and fixed in 4% formaldehyde for 30 min. Cell senescence was assessed using a β-Galactosidase Staining Kit (Cell Signaling Technology, 9860) according to the manufacturer’s instructions. The percentage of senescent cells was determined using ImageJ software.

### Statistical Analysis

Data were analyzed by unpaired, two-tailed, Student’s *t*-tests or one-way or two-way analysis of variance (ANOVA) followed by Bonferroni’s post-test using GraphPad Prism 7.0 software. All data are presented as means ± SEM. A *p*-value < 0.05 was considered statistically significant.

## Results

### CGRP Levels Were Decreased in the Bone Marrow Supernatant of Mice During Aging

**FIGURE 1:**
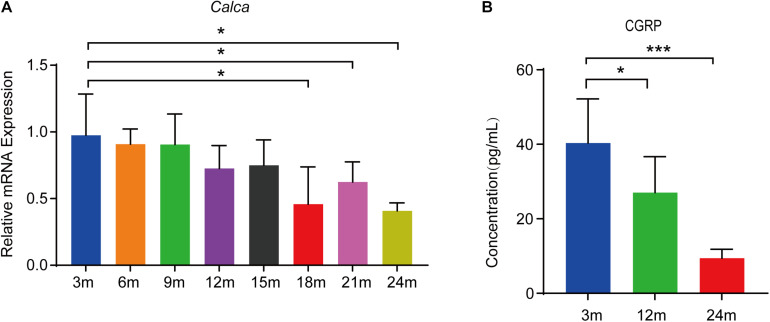
CGRP levels were decreased in the bone marrow supernatant of mice during aging. **(A)** RT-qPCR analysis of the mRNA level of *Calca* in bone tissue of mice at 3, 6, 9, 12, 15, 18, 21, and 24 months of age (*n* = 5 per age group). **(B)** The relative levels of CGRP in bone marrow supernatant were measured by ELISA. *P < 0.05, ***P < 0.001.

CGRP is a 37-residue neuropeptide primarily expressed in the central and peripheral nervous systems (Emeson et al., 1989, 1992). That CGRP plays a vital role in bone metabolism is supported by evidence showing that the lack of CGRP results in reduced bone formation and impaired bone regeneration in mice (Schinke et al., 2004; Appelt et al., 2020). To assess the expression of *Calca* (encoding α-CGRP) in bone tissue during aging, we conducted RT-qPCR analysis on total RNA extracted from the bone tissue of mice at 3, 6, 9, 12, 15, 18, 21, and 24 months of age (*n* = 5 per age group). We found that the *Calca* level was significantly decreased during aging (Figure 1A). CGRP is thought to be a secreted neuropeptide that is released from sensory nerve endings (Tsujikawa et al., 2007). To assess the CGRP levels in bone marrow, we collected bone marrow supernatant from male C57BL/6JN mice aged 3, 12, and 24 months (*n* = 5 per age group) and measured CGRP levels by ELISA. As shown in Figure 1B, CGRP levels were lower in aged mice than in young mice. Given the vital role of CGRP in bone metabolism (Irie et al., 2002; Schinke et al., 2004; Appelt et al., 2020) and that CGRP has been reported to stimulate the proliferation and osteogenic differentiation of rat-derived BMSCs (Liang et al., 2015), we subsequently hypothesized that CGRP may be involved in regulating BMSC functions during the aging process in mice.

### CGRP Treatment Promoted BMSC Osteogenic Differentiation and Reduced BMSC Senescence

**FIGURE 2:**
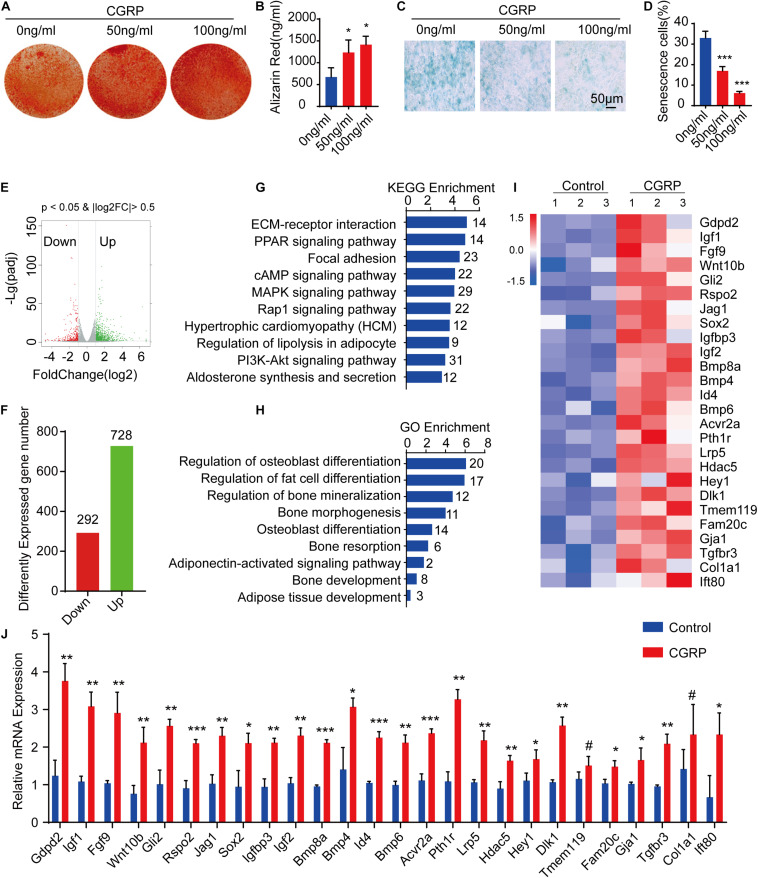
CGRP induced the osteogenic differentiation of bone marrow-derived stem cells (BMSCs) and reduced BMSC senescence. **(A)** Representative images of Alizarin Red staining and **(B)** quantitative analysis of matrix mineralization in BMSCs treated with or without CGRP. **(C)** Representative images of beta-galactosidase staining and **(D)** quantitative analysis of the percentage of senescent cells in BMSCs treated with or without CGRP. **(E)** Volcano map and **(F)** histogram of differentially expressed genes between the CGRP treatment and control groups. Red spots represent downregulated genes and green spots represent upregulated genes. **(G)** Enrichment of differentially expressed genes was tested using Kyoto Encyclopedia of Genes and Genomes (KEGG) pathway analysis and **(H)** Gene Ontology (GO) analysis. **(I)** A heatmap of the mRNA-seq profile of BMSCs treated with or without CGRP. Fold-change ≥ 2.0. **(J)** RT-qPCR analysis of gene expression levels in BMSCs treated with or without CGRP. Scale bar, 50 μm. Data are presented as means ± SEM. *P < 0.05, **P < 0.01,***P < 0.001.

To evaluate the effect of CGRP on the osteogenic differentiation potential of BMSCs, we isolated BMSCs from femoral and tibial bone marrow of mice and cultured them first in a complete medium, and then in an osteogenesis induction medium containing CGRP at 0, 50, or 100 ng/mL. Alizarin Red staining and quantitative analysis of calcium content indicated that the ability of BMSCs to form mineralized nodules was enhanced in the CGRP treatment group compared with that in the control group (Figures 2A,B). A similar result was obtained for BMSCs transfected with a CGRP expression plasmid (Supplementary Figures 1A,B). In mice, the capacity of BMSCs to differentiate into osteoblasts is known to decrease with age. Consequently, we next measured the effect of CGRP on BMSC senescence by β-galactosidase staining. As expected, the percentage of senescent cells (β-galactosidase^+^) was significantly lower in the CGRP treatment group than in the control group (Figures 2C,D). To determine the mechanism underlying the effect of CGRP on osteogenic differentiation of BMSCs and BMSC senescence, we performed RNA-seq to identify differences in mRNA expression levels between the CGRP treatment group and the control group. A total of 1,020 differentially expressed mRNAs (log2 fold-change ≥ 2) were identified (Figures 2E,F). Using Kyoto Encyclopedia of Genes and Genomes (KEGG) pathway and Gene Ontology (GO) enrichment analyses of the differentially expressed genes (Figures 2G,H), we identified several biological processes involved in osteogenic and fat cell differentiation (Figure 2H). Analysis of a heatmap depicting the differentially expressed genes involved in the regulation of osteoblast differentiation demonstrated that almost all of these genes, including *Gdpd2*, *Igf1*, *Fgf9*, *Wnt10b*, *Gli2*, *Rspo2*, *Jag1*, *Sox2*, *Igfbp3*, *Igf2*, *Bmp4*, *Bmp6*, *Bmp8a*, *Id4*, *Acvr2a*, *Pth1r*, *Lrp5*, *Hdac5*, *Hey1*, *Dlk1*, *Tmem119*, *Fam20c*, *Gja1*, *Tgfbr3*, *Col1a1*, and *Ift80*, were markedly upregulated in the CGRP treatment group compared with that in the control group (Figure 2I). The result of the RNA-seq was further confirmed by RT-qPCR analysis (Figure 2J). Combined, the results showed that CGRP treatment dose-dependently increased the osteogenic differentiation potential of BMSCs and reduced BMSC senescence.

### CGRP Inhibited the Adipogenic Differentiation of BMSCs

**FIGURE 3:**
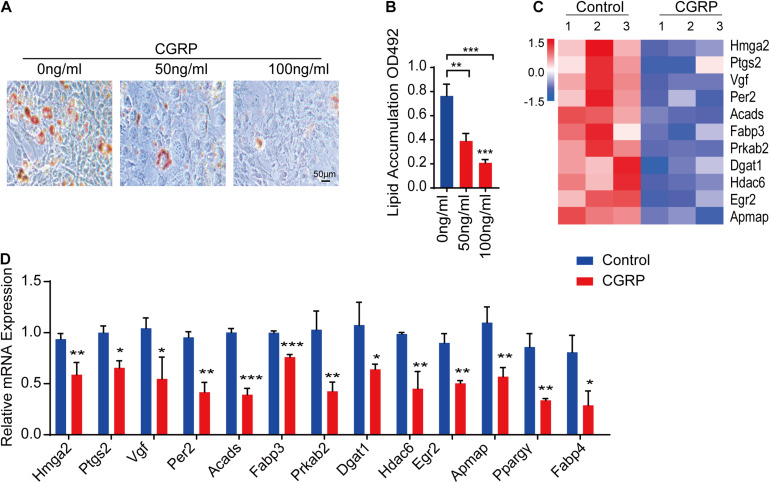
CGRP inhibited the adipogenic differentiation of bone marrow-derived stem cells (BMSCs). **(A)** Representative images of Oil Red O staining and **(B)** quantitative analysis of lipid droplet formation in BMSCs treated with or without CGRP. **(C)** A heatmap of the mRNA-seq profile of BMSCs treated with or without CGRP. Fold change ≥ 2.0. **(D)** RT-qPCR analysis of gene expression levels in BMSCs treated with or without CGRP. Scale bar, 50 μm. Data are presented as means ± SEM. *P < 0.05, **P < 0.01,***P < 0.001.

BMSCs are multipotent stem cells capable of differentiating into multiple lineages, including osteoblasts and adipocytes (Pittenger et al., 1999; James, 2013). The differentiation potential of BMSCs is influenced by multiple factors, such as injury. Given our above results showing that CGRP can induce the osteogenic differentiation of BMSCs and reduce BMSC senescence, we then tested whether CGRP treatment could also affect the adipogenic differentiation capacity of BMSCs. To evaluate this possibility *in vitro*, BMSCs were cultured in an adipogenesis induction medium containing CGRP at 0, 50, or 100 ng/mL. Oil Red O staining results showed that there were fewer lipid droplets and their size was reduced in CGRP-treated BMSCs compared with that in control BMSCs (Figures 3A,B). We obtained a similar result in BMSCs transfected with a CGRP expression plasmid (Supplementary Figures 1C,D). Next, we identified genes involved in promoting adipogenesis that were differentially expressed between the CGRP treatment group and the control group. Heatmap analysis showed that, compared with control BMSCs, the expression of *Hmga2*, *Ptgs2*, *Vgf*, *Per2*, *Acads*, *Fabp3*, *Prkab2*, *Dgat1*, *Hdac6*, *Egr2*, and *Apmap* was markedly downregulated in those treated with CGRP (Figure 3C). RT-qPCR analysis further confirmed the RNA-seq results (Figure 3D). These data indicated that CGRP treatment downregulated the expression of adipogenesis-related genes and inhibited the adipogenic differentiation capacity of BMSCs.

### CGRP Treatment Promoted Bone Formation in Aged Mice

**FIGURE 4:**
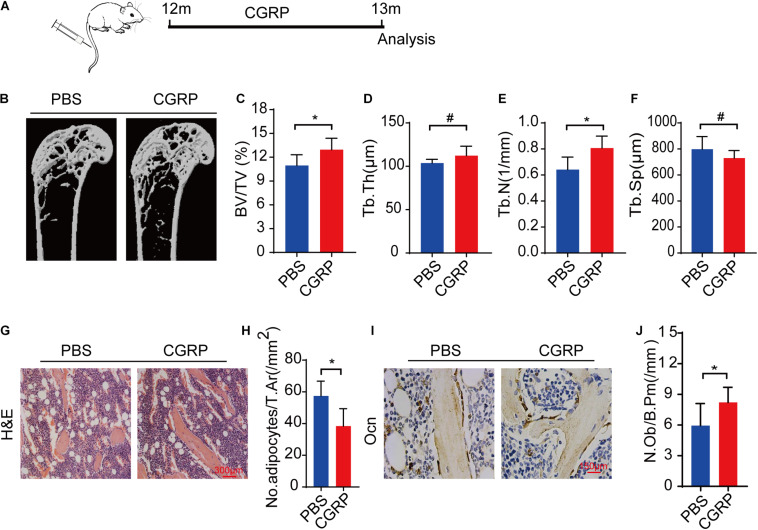
CGRP treatment promoted bone formation in aged mice. **(A)** A schematic representation of the injection protocol (CGRP or 1 × PBS *via* the tail vein) in aged mice. **(B)** Representative micro-computed tomographs (*n* = 6/group) and **(C–F)** quantitative analysis of bone volume as a fraction of total bone volume (BV/TV), trabecular thickness (Tb. Th), trabecular number (Tb. N), and trabecular separation (Tb. Sp) of femora from CGRP-treated and control mice. **(G)** Representative images of hematoxylin and eosin staining and **(H)** quantitative analysis of the number and area of adipocytes in the bone marrow of CGRP-treated and control mice. **(I)** Representative images of osteocalcin (OCN) staining and **(J)** quantification of osteoblast bone surface density (N.Ob/B.Pm) in the femora of CGRP-treated and control mice. Scale bars, 150 μm. Data are presented as means ± SEM. *P < 0.05. # means no statistical significance.

To evaluate whether CGRP could promote bone formation in aged mice *in vivo*, 12-month-old male C57BL/6JN mice (*n* = 5) were administered CGRP (10 mg/kg) or 1 × PBS *via* tail vein injection three times weekly (Figure 4A). After 1 month, the bone phenotype of the mice in both groups was analyzed. The results showed that bone mass, BV, Tb. Th, and Tb. N were markedly higher, and the Tb. Sp lower, in mice treated with CGRP when compared with those treated with PBS (Figures 4B–F). Hematoxylin and eosin (H&E) staining results showed that, compared with PBS-treated mice, those treated with CGRP had fewer adipocytes, and the area occupied by them was smaller (Figures 4G,H). Moreover, the number of osteocalcin^+^ osteoblasts on the trabecular surface was also higher in mice treated with CGRP compared with that of the control group (Figures 4I,J). These results suggested that CGRP treatment induced osteoblast differentiation and promoted bone formation in aged mice.

### CGRP Treatment Promoted Bone Formation in OVX Mice

**FIGURE 5:**
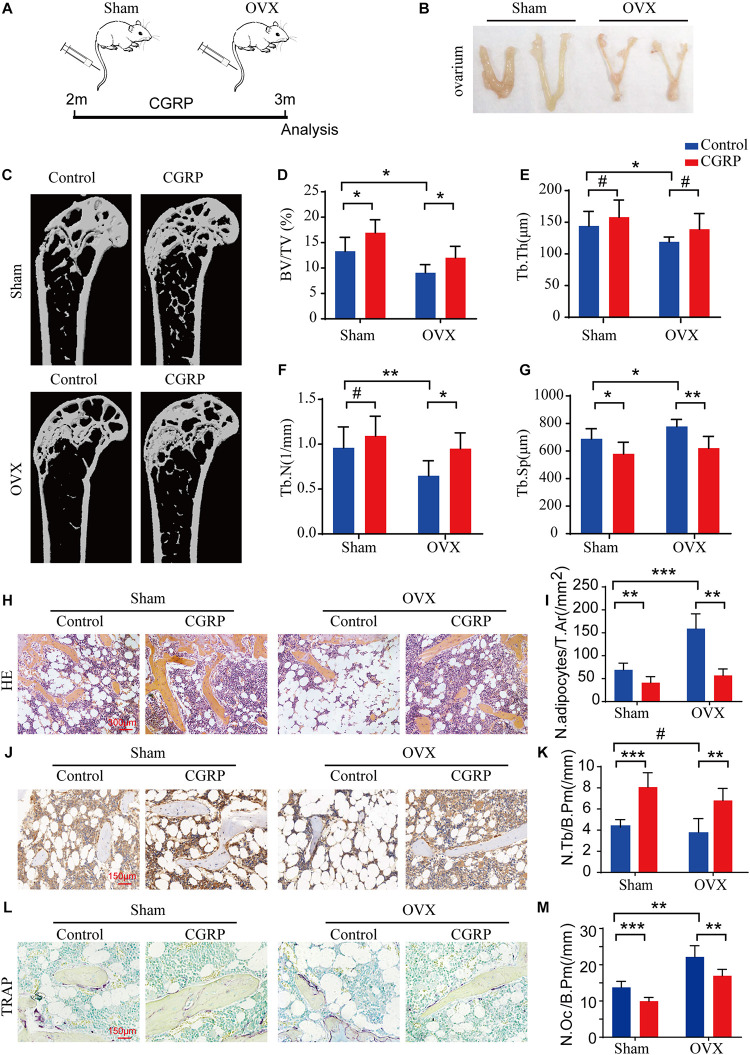
CGRP treatment promoted bone formation in ovariectomized (OVX) mice. **(A)** A schematic representation of the injection protocol (CGRP or 1 × PBS *via* the tail vein) for OVX and control (sham-operated) mice. **(B)** Representative images of the ovaries of OVX and control (sham-operated) mice treated with or without CGRP. **(C)** Representative micro-computed tomographs (*n* = 6/group) and **(D–G)** quantitative analysis of bone volume as a fraction of total bone volume (BV/TV), trabecular thickness (Tb. Th), trabecular number (Tb. N), and trabecular separation (Tb. Sp) of femora from OVX and control (sham-operated) mice treated with or without CGRP. **(H)** Representative images of hematoxylin and eosin staining and **(I)** quantitative analysis of the number and area of adipocytes in the bone marrow of OVX and control (sham-operated) mice treated with or without CGRP. Scale bar, 300 μm. **(J)** Representative images of osteocalcin (OCN) staining and **(K)** quantification of osteoblast bone surface density (N.Ob/B.Pm) in the femora of OVX and control (sham-operated) mice treated with or without CGRP. **(L)** Representative images of tartrate-resistant acid phosphatase (TRAP) staining and **(M)** quantification of osteoclast bone surface density (N.Oc/B.Pm) in the femora of OVX and control (sham-operated) mice treated with or without CGRP. Scale bar, 150 μm. Data are presented as means ± SEM. *P < 0.05, **P < 0.01,***P < 0.001. # means no statistical significance.

The OVX rodent model is well-established as a means for investigating osteoporosis and osteoporotic therapies (Mathavan et al., 2015). Here, to test the effect of CGRP on bone formation in OVX mice, we generated a model of postmenopausal osteoporosis *via* bilateral ovarian resection in 2-month-old female C57BL/6JN mice (*n* = 12/group). One month after the operation, CGRP (10 mg/kg) or 1 × PBS (10 mg/kg) was administered into mice of the OVX and sham operation groups *via* tail vein injection and ovarian injections three times weekly (Figures 5A,B). We found that the BV and Tb. Th were markedly reduced in the OVX group compared with those in the sham operation group, indicating that the model of postmenopausal osteoporosis had been successfully generated (Figures 5C–G). Moreover, bone mass, BV, Tb. Th, and Tb. N were markedly increased, while the Tb. Sp. was reduced, in CGRP-treated mice from both the OVX and sham operation groups compared with that in PBS-treated mice from both groups (Figure 5C–G). Additionally, when compared with control mice, CGRP-treated mice from the OVX and sham operation groups displayed fewer adipocytes and smaller adipocyte-containing areas in the bone marrow (Figures 5H,I), as well as greater numbers of osteocalcin^+^ osteoblasts and alkaline phosphatase^+^ osteoprogenitors on the bone surfaces (Figures 5J,K). In contrast, CGRP-treated mice from the OVX and sham operation groups had fewer TRAP^+^ osteoclasts on the bone surfaces (Figures 5L,M), which was consistent with the results of previous *in vitro* studies (Wang et al., 2010). Taken together, these results suggested that CGRP can promote bone formation in both aged and osteoporotic (OVX) mice, and suggest a potential approach for the treatment of age-related osteoporosis.

## Discussion

Osteoporosis is a chronic, age-related disease that seriously affects the quality of life of both the elderly and postmenopausal women (Rachner et al., 2011). Owing to the aging of the world’s population, it is increasingly important to understand the role of neuropeptides and their receptors in the aging process (Naot and Cornish, 2008). In the elderly, BMSCs have a greater capacity to differentiate along adipocytic lineages than along osteoblastic lineages, leading to the gradual accumulation of fat and the loss of bone (Bartel, 2004). Here, we demonstrated that CGRP is involved in the shift in BMSC cell lineage commitment, which leads to increased osteogenic differentiation and reduced adipogenic differentiation of BMSCs. Furthermore, we demonstrated for the first time that systemic CGRP administration can reduce fat accumulation, as well as promote bone formation, in both aged and OVX mice, likely by acting on BMSCs.

CGRP can reportedly regulate BMSC lineage commitment (Villa et al., 2003; Wang et al., 2010; Naot et al., 2019). In addition, several studies have shown that CGRP levels are influenced by age, and that these age-related changes can affect osteogenesis (Imai and Matsusue, 2002; Villa et al., 2003; Niedermair et al., 2020). These results are consistent with those reported here. We found that the level of CGRP in bone marrow supernatants was inversely proportional to age. Furthermore, CGRP treatment enhanced the ability of BMSCs to form mineralized nodules and significantly reduced the percentage of senescent BMSCs. Given the interaction between osteogenesis and adipogenesis, we further examined the role of CGRP in adipogenic differentiation, and report for the first time that CGRP can inhibit the adipogenic differentiation of BMSCs. These results indicated that CGRP regulates BMSC lineage commitment during aging and contributes to age-related bone formation.

It has recently been suggested that CGRP is a key neuropeptide in bone metabolism. Niedermair et al. (2020) demonstrated that CGRP regulates the bone remodeling properties of osteoblasts and osteoclasts in an age-dependent manner. Further, Mi et al. (2021) reported that CGRP can increase the endothelial progenitor cell population in the endothelial differentiation of BMSCs *in vitro*, thereby promoting bone regeneration in a rat model of distraction osteogenesis. In addition, it has been shown that CGRP can enhance BMP2 signal transduction, the expression of related osteogenic genes, and mineralization *in vitro* (Tuzmen and Campbell, 2018). He et al. (2016) reported that CGRP can maintain bone mass by stimulating osteoblast differentiation and inhibiting RANKL-induced osteoclastogenesis and bone resorption. CGRP has also been indicated to serve as a protective mechanism against particle-induced osteolysis (Kauther et al., 2011). However, to date, no work has reported on the role of CGRP in the regulation of BMSC function. In the present study, we defined a new mechanism through which CGRP regulates the BMSC switch in the bone. We further identified biological processes involved in both osteogenic and adipocyte differentiation and found that CGRP treatment led to a significant upregulation of the expression of almost all osteogenesis-related genes. Furthermore, based on the decrease in the expression level of adipogenesis-related genes, we reasoned that CGRP might suppress adipogenesis.

These results suggested that CGRP influences the direction of BMSC differentiation by regulating the expression of related genes, thereby providing a neuropeptide-mediated link to the age-related transition between osteoblast and adipocyte differentiation. During the aging process, the level of CGRP and the expression of osteogenic-related genes in the bone marrow decreases, whereas that of lipid-related genes increases. Consequently, BMSCs tend to differentiate into adipocytes, leading to a reduction in the number of osteoblasts and an increase in that of adipocytes, resulting in age-related bone loss. Here, we identified a new mechanism through which CGRP regulates the differentiation of BMSCs during aging. Relatively few studies have investigated the role of CGRP in bone metabolism *in vivo*. Here, *via* the delivery of CGRP to BMSCs by tail vein injection, we confirmed *in vivo* that CGRP can promote the osteogenic differentiation of BMSCs and inhibit fat accumulation in the bone marrow. The results of bone histomorphometry in the CGRP-treated group were significantly better than those in the control group in both aged and OVX mice. *In vitro* and *in vivo* experiments both confirmed that CGRP can promote the osteogenic differentiation and inhibit the adipogenic differentiation of BMSCs.

With the aging of the world’s population, age-related osteoporosis has become a serious public health issue (Curtis and Safford, 2012). However, most drugs currently used to treat osteoporosis are aimed at inhibiting bone resorption without promoting bone formation, and most are accompanied by severe side effects (Hu et al., 2020). In this study, we identified a new neuropeptide that may serve as potential therapeutic target for the treatment of osteoporosis. To date, CGRP has been applied in the treatment of several diseases, such as migraine, diabetes, and liver damage, as well as for cardioprotection (Kroeger et al., 2009; Yuan et al., 2017; Guo et al., 2018, 2020), suggesting that CGRP can be used safely and effectively in the treatment of age-related osteoporosis.

## Conclusion

In conclusion, our findings support that the age-related changes in CGRP levels regulate BMSC differentiation. Our results revealed a novel mechanism underlying age-related bone loss and provide a potential therapeutic strategy to treat age-related osteoporosis.

## Data Availability Statement

The raw data supporting the conclusions of this article will be made available by the authors, without undue reservation.

## Ethics Statement

All animal care protocols and experiments were reviewed and approved by the Animal Care and Use Committee of the Laboratory Animal Research Center at the Second Xiangya Hospital of Central South University. All mice were maintained in the specific pathogen–free facility of the Laboratory Animal Research Center at Central South University.

## Author Contributions

QL conceived the study. HL and RW drafted the manuscript. HZ and JW designed the figures. XX and JQ designed the tables. QL and RW revised the manuscript. All the authors were involved in the critical revision of the manuscript and approved the final version.

## Conflict of Interest

The authors declare that the research was conducted in the absence of any commercial or financial relationships that could be construed as a potential conflict of interest.

## References

[B1] Amara S. G. Jonas V. Rosenfeld M. G. Ong E. S. Evans R. M. (1982). Alternative RNA processing in calcitonin gene expression generates mRNAs encoding different polypeptide products. *Nature* 298 240–244. 10.1038/298240a0 6283379

[B2] Appelt J. Baranowsky A. Jahn D. Yorgan T. Kohli P. Otto E. (2020). The neuropeptide calcitonin gene-related peptide alpha is essential for bone healing. *EBioMedicine* 59:102970. 10.1016/j.ebiom.2020.102970 32853990 PMC7452713

[B3] Bartel D. P. (2004). MicroRNAs: genomics, biogenesis, mechanism, and function. *Cell* 116 281–297. 10.1016/s0092-8674(04)00045-5 14744438

[B4] Bjurholm A. Kreicbergs A. Brodin E. Schultzberg M. (1988). Substance P- and CGRP-immunoreactive nerves in bone. *Peptides* 9 165–171. 10.1016/0196-9781(88)90023-x 2452430

[B5] Chattergoon N. N. D’Souza F. M. Deng W. Chen H. Hyman A. L. Kadowitz P. J. (2005). Antiproliferative effects of calcitonin gene-related peptide in aortic and pulmonary artery smooth muscle cells. *Am. J. Physiol. Lung Cell Mol. Physiol.* 288 L202–L211. 10.1152/ajplung.00064.2004 15257984

[B6] Chen X. Zhi X. Wang J. Su J. (2018). RANKL signaling in bone marrow mesenchymal stem cells negatively regulates osteoblastic bone formation. *Bone Res.* 6:34. 10.1038/s41413-018-0035-6 30510839 PMC6255918

[B7] Childs B. G. Durik M. Baker D. J. van Deursen J. M. (2015). Cellular senescence in aging and age-related disease: from mechanisms to therapy. *Nat. Med.* 21 1424–1435. 10.1038/nm.4000 26646499 PMC4748967

[B8] Curtis J. R. Safford M. M. (2012). Management of osteoporosis among the elderly with other chronic medical conditions. *Drugs Aging* 29 549–564. 10.2165/11599620-000000000-00000 22715862 PMC3767038

[B9] Emeson R. B. Hedjran F. Yeakley J. M. Guise J. W. Rosenfeld M. G. (1989). Alternative production of calcitonin and CGRP mRNA is regulated at the calcitonin-specific splice acceptor. *Nature* 341 76–80. 10.1038/341076a0 2788825

[B10] Emeson R. B. Yeakley J. M. Hedjran F. Merillat N. Lenz H. J. Rosenfeld M. G. (1992). Posttranscriptional regulation of calcitonin/CGRP gene expression. *Ann. N. Y. Acad. Sci.* 657 18–35. 1386208

[B11] Guo Y. Chen H. Jiang Y. Yuan Y. Zhang Q. Guo Q. (2020). CGRP regulates the dysfunction of peri-implant angiogenesis and osseointegration in streptozotocin-induced diabetic rats. *Bone* 139:115464. 10.1016/j.bone.2020.115464 32504826

[B12] Guo Z. Liu N. Chen L. Zhao X. Li M. R. (2018). Independent roles of CGRP in cardioprotection and hemodynamic regulation in ischemic postconditioning. *Eur. J. Pharmacol.* 828 18–25. 10.1016/j.ejphar.2018.03.031 29572067

[B13] He H. Chai J. Zhang S. Ding L. Yan P. Du W. (2016). CGRP may regulate bone metabolism through stimulating osteoblast differentiation and inhibiting osteoclast formation. *Mol. Med. Rep.* 13 3977–3984. 10.3892/mmr.2016.5023 27035229

[B14] Hu Q. Long C. Wu D. You X. Ran L. Xu J. (2020). The efficacy and safety of ipriflavone in postmenopausal women with osteopenia or osteoporosis: a systematic review and meta-analysis. *Pharmacol. Res.* 159:104860. 10.1016/j.phrs.2020.104860 32407952

[B15] Idris A. I. Sophocleous A. Landao-Bassonga E. Canals M. Milligan G. Baker D. (2009). Cannabinoid receptor type 1 protects against age-related osteoporosis by regulating osteoblast and adipocyte differentiation in marrow stromal cells. *Cell Metab.* 10 139–147. 10.1016/j.cmet.2009.07.006 19656492

[B16] Imai S. Matsusue Y. (2002). Neuronal regulation of bone metabolism and anabolism: calcitonin gene-related peptide-, substance P-, and tyrosine hydroxylase-containing nerves and the bone. *Microsc. Res. Tech.* 58 61–69. 10.1002/jemt.10119 12203704

[B17] Imai S. Rauvala H. Konttinen Y. T. Tokunaga T. Maeda T. Hukuda S. (1997). Efferent targets of osseous CGRP-immunoreactive nerve fiber before and after bone destruction in adjuvant arthritic rat: an ultramorphological study on their terminal-target relations. *J. Bone Miner Res.* 12 1018–1027. 10.1359/jbmr.1997.12.7.1018 9200000

[B18] Irie K. Hara-Irie F. Ozawa H. Yajima T. (2002). Calcitonin gene-related peptide (CGRP)-containing nerve fibers in bone tissue and their involvement in bone remodeling. *Microsc. Res. Tech.* 58 85–90. 10.1002/jemt.10122 12203707

[B19] James A. W. (2013). Review of signaling pathways governing MSC osteogenic and adipogenic differentiation. *Scientifica (Cairo)* 2013 684736. 10.1155/2013/684736 24416618 PMC3874981

[B20] Kauther M. D. Bachmann H. S. Neuerburg L. Broecker-Preuss M. Hilken G. Grabellus F. (2011). Calcitonin substitution in calcitonin deficiency reduces particle-induced osteolysis. *BMC Musculoskelet Disord.* 12:186. 10.1186/1471-2474-12-186 21843355 PMC3171722

[B21] Kroeger I. Erhardt A. Abt D. Fischer M. Biburger M. Rau T. (2009). The neuropeptide calcitonin gene-related peptide (CGRP) prevents inflammatory liver injury in mice. *J. Hepatol.* 51 342–353. 10.1016/j.jhep.2009.03.022 19464067

[B22] Li C. J. Cheng P. Liang M. K. Chen Y. S. Lu Q. Wang J. Y. (2015). MicroRNA-188 regulates age-related switch between osteoblast and adipocyte differentiation. *J. Clin. Invest.* 125 1509–1522. 10.1172/JCI77716 25751060 PMC4396470

[B23] Li H. Liu P. Xu S. Li Y. Dekker J. D. Li B. (2017). FOXP1 controls mesenchymal stem cell commitment and senescence during skeletal aging. *J. Clin. Invest.* 127 1241–1253. 10.1172/JCI89511 28240601 PMC5373872

[B24] Li K. C. Chang Y. H. Yeh C. L. Hu Y. C. (2016). Healing of osteoporotic bone defects by baculovirus-engineered bone marrow-derived MSCs expressing MicroRNA sponges. *Biomaterials* 74 155–166. 10.1016/j.biomaterials.2015.09.046 26454414

[B25] Liang W. Zhuo X. Tang Z. Wei X. Li B. (2015). Calcitonin gene-related peptide stimulates proliferation and osteogenic differentiation of osteoporotic rat-derived bone mesenchymal stem cells. *Mol. Cell Biochem.* 402 101–110. 10.1007/s11010-014-2318-6 25563479

[B26] Lv Y. J. Yang Y. Sui B. D. Hu C. H. Zhao P. Liao L. (2018). Resveratrol counteracts bone loss via mitofilin-mediated osteogenic improvement of mesenchymal stem cells in senescence-accelerated mice. *Theranostics* 8 2387–2406. 10.7150/thno.23620 29721087 PMC5928897

[B27] Mathavan N. Turunen M. J. Tagil M. Isaksson H. (2015). Characterising bone material composition and structure in the ovariectomized (OVX) rat model of osteoporosis. *Calcif. Tissue Int.* 97 134–144. 10.1007/s00223-015-9991-7 25894067

[B28] Mi J. Xu J. Yao H. Li X. Tong W. Li Y. (2021). Calcitonin gene-related peptide enhances distraction osteogenesis by increasing angiogenesis. *Tissue Eng. Part A* 27 87–102. 10.1089/ten.TEA.2020.0009 32375579

[B29] Naot D. Cornish J. (2008). The role of peptides and receptors of the calcitonin family in the regulation of bone metabolism. *Bone* 43 813–818. 10.1016/j.bone.2008.07.003 18687416

[B30] Naot D. Musson D. S. Cornish J. (2019). The activity of peptides of the calcitonin family in bone. *Physiol. Rev.* 99 781–805. 10.1152/physrev.00066.2017 30540227

[B31] Niedermair T. Schirner S. Lasheras M. G. Straub R. H. Grassel S. (2020). Absence of alpha-calcitonin gene-related peptide modulates bone remodeling properties of murine osteoblasts and osteoclasts in an age-dependent way. *Mech. Ageing Dev.* 189:111265. 10.1016/j.mad.2020.111265 32446790

[B32] NIH Consensus Development Panel on Osteoporosis Prevention, Diagnosis, and Therapy (2001). Osteoporosis prevention, diagnosis, and therapy. *JAMA* 285 785–795. 10.1001/jama.285.6.785 11176917

[B33] Peng H. Yang M. Guo Q. Su T. Xiao Y. Xia Z. Y. (2019). Dendrobium officinale polysaccharides regulate age-related lineage commitment between osteogenic and adipogenic differentiation. *Cell Prolif.* 52:e12624. 10.1111/cpr.12624 31038249 PMC6668967

[B34] Picke A. K. Campbell G. M. Bluher M. Krugel U. Schmidt F. N. Tsourdi E. (2018). Thy-1 (CD90) promotes bone formation and protects against obesity. *Sci. Transl. Med.* 10:eaao6806. 10.1126/scitranslmed.aao6806 30089635

[B35] Pittenger M. F. Mackay A. M. Beck S. C. Jaiswal R. K. Douglas R. Mosca J. D. (1999). Multilineage potential of adult human mesenchymal stem cells. *Science* 284 143–147. 10.1126/science.284.5411.143 10102814

[B36] Rachner T. D. Khosla S. Hofbauer L. C. (2011). Osteoporosis: now and the future. *Lancet* 377 1276–1287. 10.1016/S0140-6736(10)62349-5 21450337 PMC3555696

[B37] Rosenfeld M. G. Mermod J. J. Amara S. G. Swanson L. W. Sawchenko P. E. Rivier J. (1983). Production of a novel neuropeptide encoded by the calcitonin gene via tissue-specific RNA processing. *Nature* 304 129–135. 10.1038/304129a0 6346105

[B38] Russell F. A. King R. Smillie S. J. Kodji X. Brain S. D. (2014). Calcitonin gene-related peptide: physiology and pathophysiology. *Physiol. Rev.* 94 1099–1142. 10.1152/physrev.00034.2013 25287861 PMC4187032

[B39] Schinke T. Liese S. Priemel M. Haberland M. Schilling A. F. Catala-Lehnen P. (2004). Decreased bone formation and osteopenia in mice lacking alpha-calcitonin gene-related peptide. *J. Bone Miner Res.* 19 2049–2056. 10.1359/JBMR.040915 15537449

[B40] Shen W. Chen J. Gantz M. Punyanitya M. Heymsfield S. B. Gallagher D. (2012). MRI-measured pelvic bone marrow adipose tissue is inversely related to DXA-measured bone mineral in younger and older adults. *Eur. J. Clin. Nutr.* 66 983–988. 10.1038/ejcn.2012.35 22491495 PMC3396793

[B41] Sternini C. (1992). Enteric and visceral afferent CGRP neurons. Targets of innervation and differential expression patterns. *Ann. N. Y. Acad. Sci.* 657 170–186. 10.1111/j.1749-6632.1992.tb22766.x 1637083

[B42] Thievent A. Sena S. Parlakian A. Breuzard G. Beley A. Rochette L. (2005). Potential role of the neuropeptide CGRP in the induction of differentiation of rat hepatic portal vein wall. *Peptides* 26 1567–1572. 10.1016/j.peptides.2005.02.015 16112394

[B43] Tsujikawa K. Yayama K. Hayashi T. Matsushita H. Yamaguchi T. Shigeno T. (2007). Hypertension and dysregulated proinflammatory cytokine production in receptor activity-modifying protein 1-deficient mice. *Proc. Natl. Acad. Sci. U.S.A.* 104 16702–16707. 10.1073/pnas.0705974104 17923674 PMC2034234

[B44] Tuzmen C. Campbell P. G. (2018). Crosstalk between neuropeptides SP and CGRP in regulation of BMP2-induced bone differentiation. *Connect Tissue Res.* 59(Suppl. 1) 81–90. 10.1080/03008207.2017.1408604 29745819 PMC6448777

[B45] Villa I. Dal Fiume C. Maestroni A. Rubinacci A. Ravasi F. Guidobono F. (2003). Human osteoblast-like cell proliferation induced by calcitonin-related peptides involves PKC activity. *Am. J. Physiol. Endocrinol. Metab.* 284 E627–E633. 10.1152/ajpendo.00307.2002 12556355

[B46] Wang L. Shi X. Zhao R. Halloran B. P. Clark D. J. Jacobs C. R. (2010). Calcitonin-gene-related peptide stimulates stromal cell osteogenic differentiation and inhibits RANKL induced NF-kappaB activation, osteoclastogenesis and bone resorption. *Bone* 46 1369–1379. 10.1016/j.bone.2009.11.029 19962460 PMC2854244

[B47] Xu Y. Xia M. Chen T. Yang Y. Fu G. Ji P. (2019). Inferior alveolar nerve transection disturbs innate immune responses and bone healing after tooth extraction. *Ann. N. Y. Acad. Sci.* 1448 52–64. 10.1111/nyas.14120 31095746

[B48] Yang M. Guo Q. Peng H. Xiao Y. Z. Xiao Y. Huang Y. (2019). Kruppel-like factor 3 inhibition by mutated lncRNA Reg1cp results in human high bone mass syndrome. *J. Exp. Med.* 216 1944–1964. 10.1084/jem.20181554 31196982 PMC6683986

[B49] Yoo Y. M. Kwag J. H. Kim K. H. Kim C. H. (2014). Effects of neuropeptides and mechanical loading on bone cell resorption in vitro. *Int. J. Mol. Sci.* 15 5874–5883. 10.3390/ijms15045874 24717410 PMC4013601

[B50] Yu B. Wang C. Y. (2016). Osteoporosis: the result of an ‘aged’ bone microenvironment. *Trends Mol. Med.* 22 641–644. 10.1016/j.molmed.2016.06.002 27354328 PMC4969144

[B51] Yuan H. Lauritsen C. G. Kaiser E. A. Silberstein S. D. (2017). CGRP monoclonal antibodies for migraine: rationale and progress. *BioDrugs* 31 487–501. 10.1007/s40259-017-0250-5 29116598

[B52] Zhang Y. Xu J. Ruan Y. C. Yu M. K. O’Laughlin M. Wise H. (2016). Implant-derived magnesium induces local neuronal production of CGRP to improve bone-fracture healing in rats. *Nat. Med.* 22 1160–1169. 10.1038/nm.4162 27571347 PMC5293535

