# Corrigendum: CGRP Regulates the Age-Related Switch Between Osteoblast and Adipocyte Differentiation

**DOI:** 10.3389/fcell.2021.715740

**Published:** 2021-06-22

**Authors:** Hang Li, Jian Qu, Haihong Zhu, Jiaojiao Wang, Hao He, Xinyan Xie, Ren Wu, Qiong Lu

**Affiliations:** ^1^Department of Pharmacy, The Second Xiangya Hospital of Central South University, Changsha, China; ^2^Institute of Clinical Pharmacy, Central South University, Changsha, China; ^3^Department of Vascular Surgery, The Second Xiangya Hospital of Central South University, Changsha, China; ^4^Department of Orthopedics, The Second Xiangya Hospital of Central South University, Changsha, China

**Keywords:** CGPR, osteoporosis, BMSCs, osteogenic, adipogenic, age-related

In the published article, there was an error regarding the affiliation(s) for Qiong Lu. It should be Qiong Lu^1, 2*^ instead of Qiong Lu^2, 3*^.

The authors apologize for this error and state that this does not change the scientific conclusions of the article in any way. The original article has been updated.

