# Supplementary material for: CGRP Regulates the Age-Related Switch Between Osteoblast and Adipocyte Differentiation
Source: Front Cell Dev Biol. 2021 May 26;9:675503. doi: 10.3389/fcell.2021.675503 (PMC8187789; doi:10.3389/fcell.2021.675503)
Supplement: Supplementary file 1 [file Data_Sheet_1.docx]

Supplementary Material

# Supplementary Figures

**
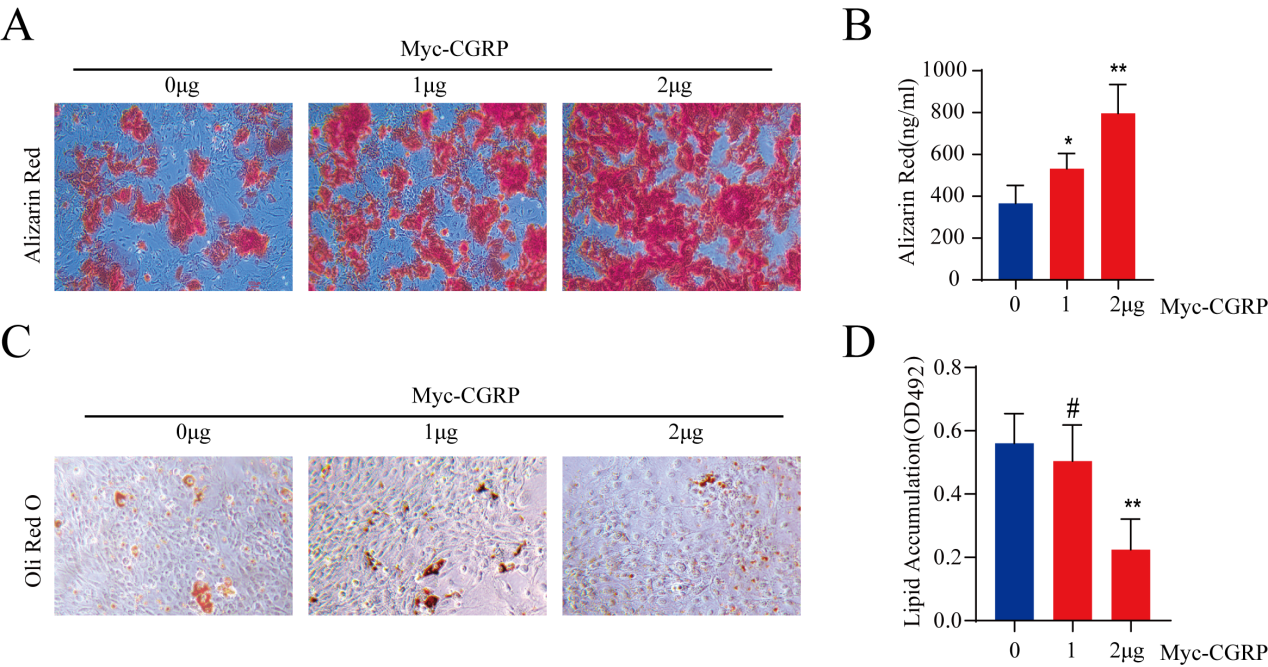
**

**Supplementary Figure 1.** The overexpression of CGRP promoted the osteogenic differentiation and inhibited the adipogenic differentiation of bone marrow-derived stem cells (BMSCs). (A) Representative images of Alizarin Red staining and (**B**) quantitative analysis of matrix mineralization. (**C**) Representative images of Oil Red O staining and (**D**) quantitative analysis of lipid droplet formation in BMSCs. Data are presented as means ± SEM. **P* < 0.05, ***P* < 0.01.
